# Suppression of monocyte and neutrophil function by recombinant IL-2

**DOI:** 10.1155/S0962935193000080

**Published:** 1993

**Authors:** Susan E. Smith, Gayle D. Warren, Yee-Hing Thong, Joe G. McCormack

**Affiliations:** 1 Department of Child Health and Immunobiology Laboratory University of Queensland Mater Misericordiae Hospitals, South Brisbane Queensland 4101 Australia; 2 Department of Medicine Immunobiology Laboratory University of Queensland Mater Misericordiae Hospitals, South Brisbane Queensland 4101 Australia

## Abstract

Little IS known about the influence of IL-2 on phagocytes. We now describe the effects of human recombinant IL-2 on human neutrophil and monocyte functions related to mobility, phagocytosis, glucose uptake, respiration and degranulation. Neutrophil adherence and hexose monophosphate shunt activities were both suppressed after incubation with IL-2. IL-2 had no effect on neutrophil migration, phagocytosis, deoxyglucose uptake or degranulation, ionocytes demonstrated a greater sensitivity to IL-2 with suppression of monocyte adherence, random and stimulated migration, glucose uptake and hexose monophosphate shunt activity, even after addition of phorbol myristate acetate. Monocyte phagocytosis and degranulation were not affected. All of the effects observed were dose-dependent within a biologically active range for IL-2. These studies suggest that IL-2 may have an important down-regulatory role across a broad range of monocyte functions including movement, deoxyglucose uptake and respiration. However, its role in regulation of neutrophil function is limited to adherence and respiration. IL-2 may be a more versatile cytokine than has previously been appreciated.


Mediators of Inflammation, 2, 59–65 (1993)

### Suppression of monocyte and neutrophil function by recombinant IL-2

Susan E. Smith,[1,2] Gayle D. Warren,[1] Yee-Hing Thong[1] and Joe G. McCormack[2,CA]

[1] Department of Child Health; and
[2] Department of Medicine, Immunobiology Laboratory, University of Queensland, Mater Misericordiae Hospitals, South Brisbane, Queensland, 4101, Australia

[CA] Corresponding Author

**LITTLE IS known about the influence of IL-2 on phagocytes. We now describe the effects of human recombinant IL-2 on human neutrophil and monocyte functions related to mobility, phagocytosis, glucose uptake, respiration and degranulation. Neutrophil adherence and hexose monophosphate shunt activities were both suppressed after incubation with IL-2. IL-2 had no effect on neutrophil migration, phagocytosis, deoxyglucose uptake or degranulation. Monocytes demonstrated a greater sensitivity to IL-2 with suppression of monocyte adherence, random and stimulated migration, glucose uptake and hexose monophosphate shunt activity, even after addition of phorbol myristate acetate. Monocyte phagocytosis and degranulation were not affected. All of the effects observed were dose-dependent within a biologically active range for IL-2. These studies suggest that IL-2 may have an important down-regulatory role across a broad range of monocyte functions including movement, deoxyglucose uptake and respiration. However, its role in regulation of neutrophil function is limited to adherence and respiration. IL-2 may be a more versatile cytokine than has previously been appreciated.**

**Key words:** Cell function, Cytokine, Immune regulation, Interleukin-2, Monocyte, Neutrophil, Phagocyte suppression

### Introduction

Interleukin-2 (IL-2) is a glycoprotein cytokine with a broad range of immunoregulatory functions. It has been shown to play a role in the differentiation and proliferation of both B- and T-lymphocytes by up- or down-regulation of various subsets;[1–4] it can directly augment the cytotoxic activity of natural killer cells and large granular lymphocytes,[5–7] but may also indirectly suppress this function.[8] IL-2 has also been implicated in monocyte/macrophage regulation as these cells have been shown to bind IL-2 via specific membrane receptors[9–11] and it has been shown to enhance monocyte cytotoxic activity[12] and induce macrophage resistance to infection.[13]

The most widely studied of the membrane receptors through which the IL-2 molecule mediates its effects is the Tac protein, which is expressed on primed or preactivated cells.[13] There are, however, a number of other cell membrane proteins on the surfaces of resting and stimulated cells, which bind or are phosphorylated by IL-2.[14–16] While resting monocytes are devoid of the Tac receptor protein, expression has been shown to occur following culture with a number of classical stimuli including lipopolysaccharide and interferon-$\gamma$.[9] Addition of IL-2 to such stimulated cells causes an augmentation of microbicidal activity.[9,10,12]

Polymorphonuclear leukocytes have not been demonstrated to display the Tac protein[10] and, consequently, possible modulation of their functions by IL-2 has not been as extensively investigated. Furthermore, neutrophils have only recently been recognized as target cells for immunoregulatory cytokines.[17,18] In the light of evidence that IL-2 modulates the activities of various cell types via pathways other than the Tac protein receptor,[3,15,16] we set out to compare the effect of IL-2 on two immunocyte subsets classically devoid of Tac protein expression: the human peripheral blood mononuclear phagocytes (monocytes) and polymorphonuclear phagocytes (neutrophils).

### Methods and Materials

*Interleukin-2:* Human recombinant IL-2 (Ala125) from an *Escherichia coli* host was manufactured by Amgen Biologicals, CA, USA and obtained through Amersham International, UK, with a specific activity of 2.6–2.8 × $10^3$ unit/$\mu$l. Purity was at least 96% and lipopolysaccharide contamination was less than 0.09 pg/ml at the highest concentration of IL-2 used under the final assay conditions. The alanine substitution for cysteine at position 125 provides an increased specific activity of up to 1 × $10^7$ unit/mg protein in the T-cell

© 1993 Rapid Communications of Oxford Ltd growth assay and improves stability. Apart from this, the biological properties of this product are essentially indistinguishable from cell culture derived material. One unit of activity is defined as the amount of IL-2 which induces 50% of maximal $^3$H thymidine incorporation by IL-2 dependent T-cells in culture (Amersham Product Technical Information). A stock IL-2 solution of 1 000 U/ml was prepared in 0.1% bovine serum albumin (BSA; Sigma Chemical Co., St Louis, MO) in Dulbecco's phosphate buffered saline (DPBS; Flow Laboratories, McLean, VA), and stored in aliquots at −70°C for up to 3 months. Concentrations of 2, 20 and 200 U/ml were used for these experiments since, classically, 2–50 U/ml is required for the maintenance of IL-2 dependent T-cell lines.

*Leukocyte preparation:* Blood collected by venepuncture from healthy volunteer donors was anticoagulated with heparin. The blood was layered over Mono Poly Resolving Medium (Flow Laboratories) and centrifuged at 400 × ***g*** for 30–40 min. Neutrophils of 97% purity were removed from the lower of the two leukocyte bands formed at the interface.[19] The upper band, containing mononuclear cells and platelets, was enriched for monocytes using preformed continuous density gradients made from Percoll (Pharmacia, South Seas) as described previously.[20] Briefly, Percoll was aseptically made up to 325 mosM with a density of 1.070 g/cm$^3$ with phosphate buffered saline and 0.1% BSA; 10 ml of the solution was spun at 21 000 × ***g*** in a Beckman Ultracentrifuge (21°C, fixed angle rotor) for 20 min. The mononuclear cell suspension, depleted of platelets by centrifugation through autologous plasma, was layered over the Percoll gradient and centrifuged at 1 000 × ***g*** for 15 min. The upper of the three bands formed was collected using a hypodermic syringe and needle and routinely yielded 70–90% monocytes. The percentage of monocytes was assessed using 0.01% Methylene Blue vital staining and the accuracy of this method was periodically checked with fluorescein-conjugated MO2 monoclonal antibody (Coulter Electronics, FL, USA) staining. Leukocytes were washed twice and resuspended in Medium 199 (M199; Commonwealth Serum Laboratories, Australia) or in DPBS as dictated by assay requirements.

*Mobility assays:* Adherence of leukocytes was measured after passage through a dacron fibre (100 μg Olympic General Products, Queensland, Australia) microcolumn as described previously.[21] Neutrophils were incubated with IL-2 for 30 min prior to a 5 min contact with the fibre at 37°C in a humidified 5% $CO_2$ atmosphere. Monocytes were in contact with the fibre microcolumn for 30 min in the presence of varying concentrations of IL-2. Adherence was expressed as

$$\frac{C_O - C_E}{C_o} \times 100\%$$

where $C_o$ is the original cell concentration and $C_E$ is the cell concentration in the effluent. All monocyte suspensions were differentially stained to exclude contaminant adherent lymphocytes from the calculation. The under-agarose migration assay[22] was used to measure random and stimulated migration using autologous serum activated by *Candida albicans* as the chemoattractant. IL-2 was incorporated into 3 ml M199 and 10% FCS and 2% agarose at the required concentrations. Triplicate wells were punched 2.5 cm apart, once the agarose had set in 60 mm diameter plastic tissue culture plates. The centre well received the cell suspension, the outer well received the chemoattractant (for stimulated migration) and the inner well received M199 (for random migration). Neutrophils were allowed to migrate for 2.5 h at 37°C in a humidified $CO_2$ atmosphere while monocytes required 18 h incubation. The distance travelled towards either well was measured with the aid of an eye piece grid on an inverted microscope.

*Phagocytosis assay:* Phagocytosis was measured by the inhibition of uridine uptake by *C. albicans*; once phagocytosed, yeast cells do not incorporate uridine.[23] Neutrophils or monocytes were preincubated with IL-2 for 15 min in M199 and 2.5% autologous serum. Live, washed *C. albicans* cells were added and the mixture incubated for 30 min at 37°C with rotational mixing. Triplicate 0.1 ml aliquots of the suspensions were dispersed in a 96-well microtitre tray and 5 μl $^{14}$C uridine (Amersham) was added to achieve a final concentration of 0.93 μCi/ml. This was incubated at 37°C for 1 h, centrifuged and measured for yeast uridine incorporated (as loss of $^{14}$C from the supernatant) by liquid scintillation counting (LSC). This value was expressed as a proportion of the value obtained for suspensions of yeast without leukocytes to give a percentage of yeast phagocytosis.

*Cell respiration assays:* Glucose transport was assayed indirectly by measuring the comparative concentrations of $^3$H-2-deoxyglucose ($^3$H-DOG; Amersham, UK) in cell suspension supernatants, as described previously.[24] Briefly, cells were incubated together with varying concentrations of IL-2, in 96-well round-bottom microtitre plates (Flow Laboratories) to which $^3$H-DOG was added to give a final concentration of 0.78 μCi/ml in a total of 0.2 ml DPBS (Flow Laboratories). After allowing uptake for 30 or 60 min at 37°C for neutrophils and monocytes, respectively, the plates were centrifuged at 800 × ***g*** for 5 min and 0.05 ml of supernatant was removed for LSC. Uptake of $^3$H-DOG was calculated as the total disintegration/ml added minus the disintegration/ml remaining in the supernatant. A standard assay for hexose monophosphate shunt (HMPS) activity which measures the conversion of D-(1-$^{14}$C) glucose (Amersham)[25] to $^{14}CO_2$ in the oxidative pathway of energy generation, was adapted to microassay form using two tube caps in small sealed jars. In one of the caps, 0.4 ml cell suspensions in DPBS containing varying concentrations of IL-2 and $^{14}$C glucose at a final concentration of 0.25 $\mu$Ci/ml were incubated at 37°C for 45 min. Then 1 M hydrochloric acid was added to drive out dissolved $^{14}CO_2$ which was captured in 0.2 ml 5 M sodium hydroxide contained in the second cap and quantitated by LSC. Phorbol myristate acetate (PMA, Sigma) was added in some experiments after preliminary incubation of cells with IL-2 for 30 min.

*Degranulation assays:* A standard optical density assay for lysozyme activity based on lysis of dried *Micrococcus lysodeiktius* (Sigma reagents) was used to gauge specific granule release in the cell supernatants. Cells were incubated in 96-well microtitre plates with IL-2 in DPBS and 1% glucose for 30 min at 37°C.[26] Beta-glucuronidase activity was used as a marker for azurophilic granule release in supernatants as for lysozyme activity and was assayed spectrophotometrically using phenolphthalein-glucuronic acid substrate (Sigma).[26] For both assays data is represented as a percentage of total cell enzyme content determined from sonicated suspensions. In some experiments the calcium ionophore $A_{23187}$ (calimycin, 7.5 mM; Sigma) was added after cells were preincubated with IL-2 for 30 min.

*Statistical analysis:* All experimental data are representative of two to five similar experiments performed in triplicate with individual standard deviations (SD) of less than 10%. Values are expressed as mean $\pm$ SD and statistical significance was measured with the Student's *t*-test.

### Results

*Mobility:* Adherence of both neutrophils and monocytes to dacron fibre was decreased following incubation with IL-2 (Fig. 1). The effect on migration under agarose differed for the two phagocyte types: neither neutrophil random nor stimulated migration was altered as a result of exposure to IL-2, while both monocyte random and stimulated migration decreased significantly with increasing concentrations of IL-2 (Fig. 2(a) and (b)). Chemotactic indices for the monocytes were calculated from the ratio of stimulated migration/random migration. The indices for the

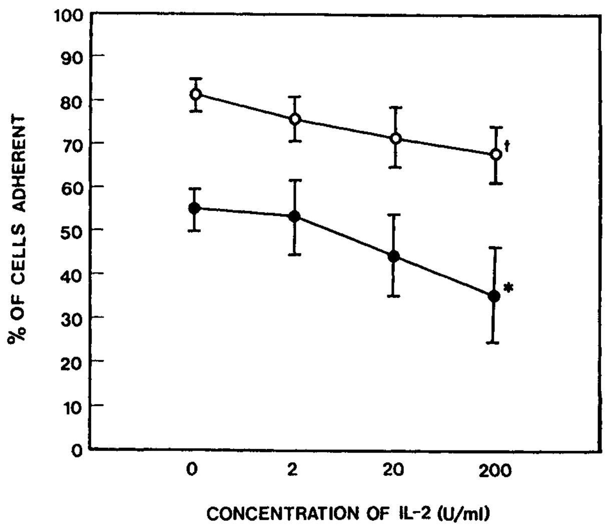

FIG. 1. Phagocyte adherence to dacron fibre decreased with increasing IL-2 concentrations. Final cell concentration was 5 × 10$^6$ ml. Neutrophils (●) and monocytes (○) both demonstrate decreased adherence at 200 U/ml IL-2. * $p \leq 0.01$, † $p \leq 0.02$.

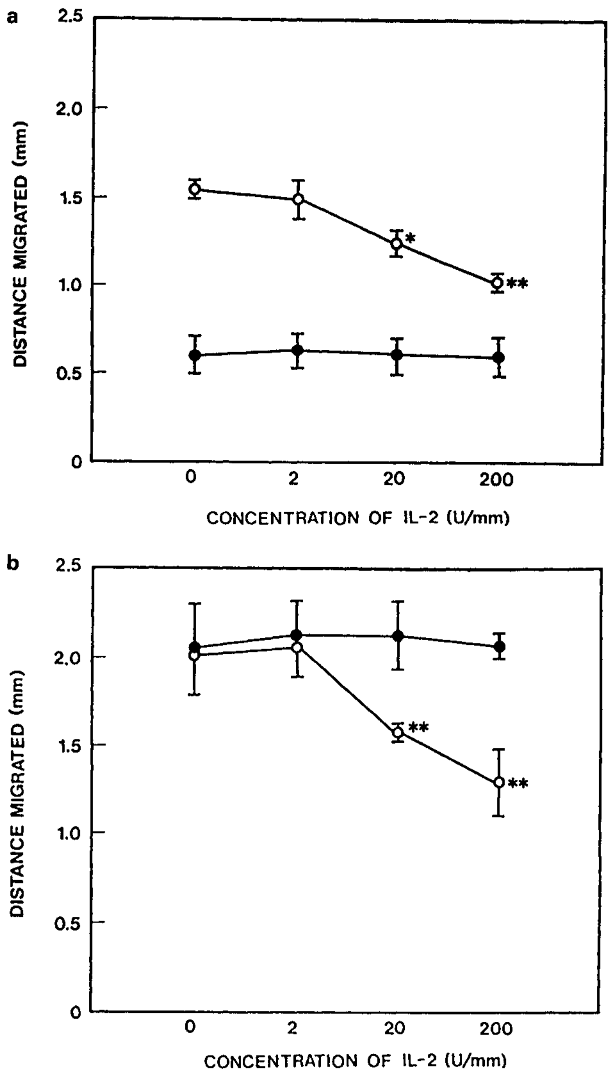

FIG. 2. (a) random and (b) stimulated migration under agarose of neutrophils (●) and monocytes (○) treated with IL-2. (Data for (a) and (b) were collected from the same experiments.) Monocyte random and stimulated migration decreased with increasing IL-2 concentrations while neutrophil migration remained unaffected. ** $p \leq 0.001$, * $p \leq 0.01$.

**Table 1.** Concentrations of IL-2 between 2 and 200 U/ml had no effect on neutrophil or monocyte phagocytosis

| [IL-2] U/ml | Neutrophils (%) | Monocytes (%) |
|---|---|---|
| 0 | 43.8 ± 4.4 | 48.8 ± 4.8 |
| 2 | 45.5 ± 5.2 | 50.0 ± 8.9 |
| 20 | 46.5 ± 6.3 | 49.0 ± 6.6 |
| 200 | 42.8 ± 6.1 | 49.8 ± 7.3 |

various concentrations of IL-2 did not vary significantly from that of the control, being 1.30 ± 0.07 for the control and 1.37 ± 0.09, 1.29 ± 0.03 and 1.29 ± 0.14 for 2, 20 and 200 U/ml, respectively.

*Phagocytosis:* Typically, under our conditions, the phagocytosis assay yielded 40–50% uptake of *C. albicans* over a 30 min incubation period, neutrophils demonstrated no response to IL-2 in this assay and monocyte phagocytosis remained similarly unaffected (Table 1).

*Respiration:* Figure 3 demonstrates that IL-2 at concentrations of 2, 20 and 200 U/ml did not significantly affect $^3$H-DOG uptake of resting neutrophils. In contrast, monocytes showed a significant decrease in transport of deoxyglucose at a concentration of 200 U/ml of IL-2 ($p < 0.01$) with individual experiments indicating significant suppression at concentrations as low as 2 U/ml (data not shown). HMPS activity was significantly depressed for neutrophils at concentrations of 20 and 200 U/ml and for monocytes at concentrations of 2, 20 and 200 U/ml (Fig. 4(a)). Stimulation with PMA at 0.1 μg/ml enhanced HMPS activity of both cells. Addition of PMA (0.1 μg/ml) to neutrophils elicited an equivalent stimulated response in both control cells (no IL-2 preincubation) and those preincubated with IL-2 at 200 U/ml. However, the addition of PMA to monocytes which had been preincubated with IL-2 induced a response significantly less than that in control monocytes (Fig. 4(b)).

*Degranulation:* Specific granule release was not significantly affected by incubation of neutrophils or monocytes with any concentration of IL-2 assessed. Similarly, azurophilic granule release did not vary significantly from the control for resting or stimulated phagocytes (Table 2(a) and (b)).

Table 3 summarizes the observed effects of IL-2 on neutrophil and monocyte function.

*Deactivated IL-2:* IL-2 at a concentration of 200 U/ml was compared with IL-2 that had been heated to 80°C for 1 h and similarly diluted. The suppression

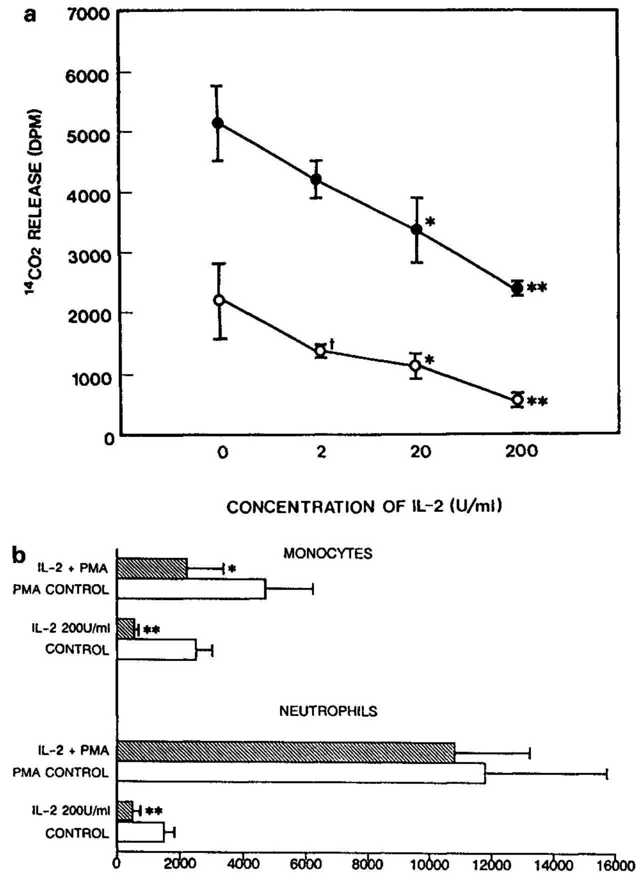

FIG. 4. (a) Effect of increasing IL-2 concentrations reduced both neutrophil (●) and monocyte (○) hexose monophosphate shunt activity. Final cell concentrations were 1 × 10$^6$ neutrophil/ml and 5 × 10$^5$ monocyte/ml. ** $p \leq 0.001$, * $p \leq 0.01$, † $p \leq 0.05$. (b) Monocytes preincubated with IL-2 at 200 U/ml for 30 min displayed reduced HMPS activity even after stimulation by PMA. Neutrophils no longer showed a significant response to IL-2 after stimulation by PMA. The control indicates the uptake of cells which did not have IL-2 added, but were otherwise treated similarly to the IL-2 test suspensions. Experimental conditions were as for (a). Statistical comparisons were made between the control/PMA control (□) and the respective IL-2 test (▨) activity, i.e. adjacent bars. * $p \leq 0.01$, ** $p \leq 0.001$.

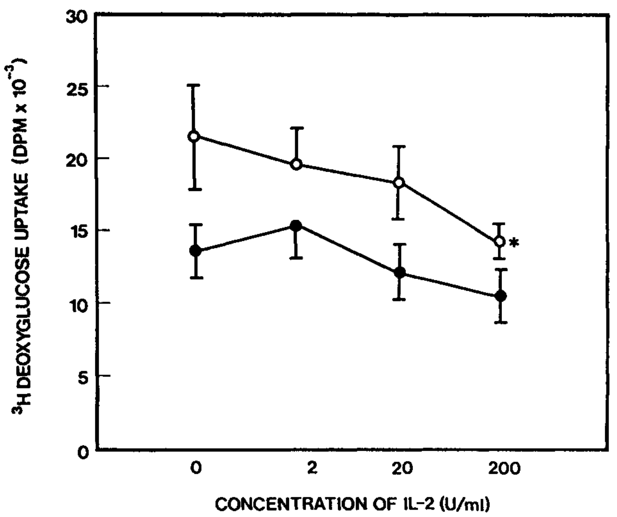

FIG. 3. Effect of increasing IL-2 concentrations on $^3$H-deoxyglucose transport. Monocyte (○) activity was reduced at 200 U/ml, neutrophil (●) activity was unaffected. Final cell concentrations were 2 × 10$^6$ neutrophil/ml and 1 × 10$^6$ monocyte/ml; $^3$H-DOG concentration was 0.78 μCi/ml. * $p \leq 0.01$.

**Table 2.** (a) Effect of IL-2 on neutrophil and monocyte azurophilic granule release[a]. (b) Effect of IL-2 on neutrophil and monocyte specific granule release with and without simulation by addition of calcium ionophore $A_{23187}$[b]

| [IL-2] | (a) Neutrophils | Monocytes | $A_{23178}$[IL-2] | (b) Neutrophils | Monocytes |
|---|---|---|---|---|---|
| 0(U/ml) | 28.2 ± 1.3 | 12.9 ± 3.5 | −[0] | 4.7 ± 1.5 | 5.2 ± 0.6 |
| 2 | 28.7 ± 2.0 | 12.3 ± 4.8 | −[2] | 4.1 ± 0.8 | 4.9 ± 0.8 |
| [20] | 25.8 ± 2.1 | 11.5 ± 4.7 | −20 | 4.4 ± 0.8 | 4.8 ± 0.4 |
| [200] | 27.4 ± 1.2 | 12.3 ± 5.7 | −[200] | 5.0 ± 0.6 | 5.1 ± 0.4 |
| | | | +[0] | 14.9 ± 4.1 | 20.3 ± 13.1 |
| | | | +[2] | 14.1 ± 2.8 | 18.3 ± 9.5 |
| | | | +20 | 14.2 ± 3.4 | 17.7 ± 10.9 |
| | | | +[200] | 16.6 ± 6.1 | 19.3 ± 10.2 |

[a] Results expressed as per cent of total β-glucuronidase content.
[b] Results expressed as per cent of total lysozyme count.

**Table 3.** Summary of assay results: effects of IL-2 on neutrophil and monocyte activities

| | Neutrophils | Monocytes |
|---|---|---|
| Adherence (Fig. 1) | Suppressed | Suppressed |
| Migration (Fig. 2) | – | Suppressed |
| Phagocytosis (Table 1) | – | – |
| Deoxyglucose uptake (Fig. 3) | – | Suppressed |
| HMP shunt (Fig. 4) | Suppressed | Suppressed |
| Degranulation (Table 2) | – | – |

of neutrophil HMPS activity and adherence by IL-2 was significantly reduced, but not totally abrogated by heating. Heat treatment also significantly reversed the suppressive effect of IL-2 on monocyte HMPS activity. The suppressive effect on monocyte migration was totally abrogated by heat treatment of IL-2. Similarly, expired IL-2 (stored at 4°C for at least 6 months) was no longer able to vary monocyte and neutrophil adherence values from that of control M199 (data not shown).

### Discussion

Under our *in vitro* conditions we have shown that recombinant IL-2 exerts a suppressive effect on selected functions of freshly isolated resting and stimulated phagocytic cells. As summarized in Table 3, neutrophil adherence was reduced while migration and phagocytosis were not. Hexose monophosphate shunt activity was reduced by IL-2, however, stimulation of the cells with PMA negated this effect. Glucose uptake and cytoplasmic granule release were not affected. Compared to neutrophils, monocyte sensitivity to IL-2 was more pronounced with both a larger number of functions being affected and more profoundly so. Monocyte adherence and migration were reduced as were glucose uptake and HMP shunt activity. Phagocytosis and granule release were not affected.

The greater diversity of monocyte functions affected by IL-2 as compared to neutrophils may not be surprising since IL-2 is classically a cytokine of the lymphoid mononuclear cell line, with which monocytes are known to interact closely in various phases of the immune response. However, the activity of IL-2 is not restricted to these cells and its effects may be diverse, for example, IL-2 is able to stimulate the release of prostacyclin by endothelial cells,[27] so again it is not unexpected that neutrophils could also be targeted.

The more profound effect of IL-2 on monocytes than on neutrophils was noticed particularly at the respiratory level. Membrane transport of deoxyglucose, a non-metabolizable glucose analogue and HMP shunt activity were both reduced in monocytes. The decreased availability of intracellular glucose correlates with the decreased activity state of the cell. As demonstrated by the migration assays, monocyte kinetic energy was reduced. The monocyte chemotactic indices (chemotaxis:random migration) for different IL-2 concentrations did not vary significantly, indicating that IL-2 did not affect the cells' ability to detect and respond to a chemotactic gradient. However, both random migration and chemotaxis values were reduced, indicating a slower response and decreased cell kinetic energy level. In contrast, neutrophil HMP shunt activity, but not deoxyglucose uptake, was suppressed by IL-2. Thus, glucose may have still been available via other respiratory pathways to support more of the neutrophils' activities.

We observed that the effect of IL-2 was not globally suppressive, but was restricted to certain areas of cell functions as has been previously noted by others. Kennedy *et al.*[1] found that IL-2 suppressed T-helper cell activation, but not T-helper cell effector function (i.e. T-helper cell–B-cell cooperation). Jablons *et al.*[28] and Klempner *et al.*[29] both reported that *in vivo* IL-2 administration resulted in depression of only some neutrophil cell functions related to host defence against bacterial infections. Jablons found neutrophil chemotaxis to be decreased together with superoxide production and FcJ 111 expression, while phagocytosis and antibody-dependent cellular cytoxicity were minimally affected.[28] Klempner *et al.*[29] also observed suppression of neutrophil chemotaxis, but found that other functions, including superoxide production were not decreased. Kowanko and Ferrante[30] have also demonstrated suppression of neutrophil migration, but stimulation of the respiratory burst and degranulation.

The contradictory effects observed by others and ourselves may be due to differences in the state of priming or deactivation of neutrophils under different study conditions.[31] Freshly harvested cells from normal donors may not be fully dormant if they have been exposed to even 'trivial' quantities of endotoxin or have been triggered by adherence to surfaces during preparation.[32] Inconsistencies in the reported effects of IL-2 may indicate that the modulatory effects of IL-2 on neutrophils are highly sensitive to the state of activation of the target cell and to the interaction of other mediators. This may be the explanation for our observation that HMP shunt activity is significantly reduced by IL-2 in resting neutrophils, but not in PMA stimulated neutrophils. Similarly the state of activation of the monocyte may alter the observed effects of IL-2. Hellstrand and Hermodsson[8] found that IL-2 induced suppression of natural killer cell cytotoxicity occurs indirectly via a p55IL-2R locus on monocytes. They showed that the primary IL-2–monocyte interaction leading to killer cell suppression could only occur in monocytes prior to adherence, demonstrating the importance of the activation state.

The variation in the observed effects of IL-2 on neutrophil chemotaxis may also be due to the different sources of IL-2 or its purity. The IL-2 Ala125 product used in this study is a recombinant molecule which contains an extra methionine residue at the N-terminus of the natural sequence molecule. This addition has had no demonstrable effect on the classic biological activity of the molecule as determined by various cell culture assay systems. The cysteine residue at position 125 has also been substituted by alanine which has resulted in a five-fold increase in specific activity as well as improving stability (Amersham Technical Information). Preliminary experiments comparing purified rat IL-2 with the Amersham product demonstrated a similar effect on neutrophil adherence (data not shown). However, the possibility remains that this difference in structure may affect the neutrophil response in the chemotaxis assay.

The effects of IL-2 on neutrophils and monocytes that we observed were consistently suppressive. Although IL-2 first became recognized because of its requirement for T-cell proliferation and, more recently, augmentation of cytotoxicity in natural killer cells and macrophages,[5–9] it has been shown to exert several down-regulatory effects, e.g. myelopoiesis, specifically granulocyte–macrophage colony formation[33,34] and T-helper cell activation.[1,4] It is unclear whether the suppressive effect of IL-2 on the myelopoietic progenitor cell is mediated via regulatory T-cells[33] or acts directly.[34] It would seem logical that any direct effect of IL-2 on the product of the myelopoietic progenitor cell, i.e. the circulating neutrophil and monocyte, may also be correspondingly suppressive under similar conditions. An indirect T-cell mediated effect is unlikely in our neutrophil experiments as the total lymphocyte contamination was only 1–2%. Burdach *et al.*[33] did observe a T-cell mediated effect, but their T-cell versus effector cell ratio was 1:5. The short assay incubation times also favour a direct effect. However, contamination of the monocyte preparation by lymphocytes ranged from 10 to 30%, which may have allowed interaction with T-cells. The assay times again favoured a direct effect in all, but the random migration and chemotaxis experiments.

Once cells migrate into tissue compartments their activation state and response may be altered, depending on the presence of other cytokines and activators. Thus, Wahl *et al.*[9] found that IL-2 augmented monocyte microbicidal activity, but only after the monocytes had been activated and cultured to express the IL-2 Tac receptor. The appearance of these receptors on the cells during the process of macrophage maturation occurs as the cells become localized in the tissues. Hall *et al.*[27] also suggested that IL-2 may regulate the inductive phase of the immune response by inhibiting the proliferative response of T-cells to mitogens and preventing further attachment of leukocytes via stimulation of prostacyclin production by endothelial cells, which may be another indirect down-regulatory effect on circulating phagocytes.

Modulation of leukocyte function by IL-2 has been demonstrated to operate via the Tac protein which was, until recently, believed to be expressed only by activated T-cells.[11] Experimental evidence has extended the modulatory role of IL-2 to include a much wider range of cells as mentioned previously. More recently, cell membrane proteins distinct from the Tac receptor protein have been found which bind IL-2 and cause activation in cells including natural killer cells and certain B-cell lines.[15] Thus, as freshly isolated peripheral blood monocytes and neutrophils do not possess the Tac IL-2 receptor protein,[10] an interaction with IL-2 via other membrane receptors is probable. Our findings support this, as the only assays we used which allow sufficient time for new Tac protein synthesis and expression were the monocyte random migration and chemotaxis assays.

We are confident that the effects we describe here are due to the activity of IL-2. We have previously demonstrated that endotoxin is unlikely to be implicated as Polymyxin B does not affect these assays[21] and the effects are depressive rather than stimulatory which would be expected of endotoxin. Furthermore, we have shown the activity to be sensitive to heat, which endotoxin is not and to fade after prolonged storage. Interestingly, the effect of IL-2 was not totally abrogated by heating as measured by the HMP shunt and adherence assays. This is consistent with the observation that the Ala125 residue substitution in our IL-2 preparation increases heat stability of the recombinant molecule over that of purified IL-2 (Amersham Technical Information). Heating of this preparation to 80°C for 24 h at the production laboratories demonstrated retention of up to 25% of original biological activity as measured by standard assay (personal communication with Amersham Technical Advisor). After 6 months storage at 4°C, IL-2 activity was totally lost as measured in the monocyte and neutrophil chemotaxis assay (data not shown). Finally, the effects observed occurred over a concentration range similar to that shown to have biological effects on other cells.

Our data supports the view that IL-2 is a multifunctional cytokine which influences a variety of immune cells and activities probably depending on the hosts' current immune state and the local tissue milieu.

### References

1. Kennedy M, Wassmer P, Erb P. A novel role for interleukin-2 in antibody responses: down-regulation of T-helper cell activation. *J Immunol* 1987; **139**: 110–113.
2. Hammer SM, Gillis JM. Effects of recombinant interleukin-2 on resting human T-lymphocytes. *J Biol Response Mod* 1989; **5**: 36–44.
3. Bich-Thuy LT, Fauci AS. Direct effect of interleukin-2 on the differentiation of human B-cells which have not been preactivated *in vitro*. *Eur J Immunol* 1985; **15**: 1075–1079.
4. Otten G, Herold KC, Fitch FW. Interleukin-2 inhibits antigen-stimulated lymphokine synthesis in helper T-cells by inhibiting calcium-dependent signalling. *J Immunol* 1987; **139**: 1348–1353.
5. Hoyer M, Meineke T, Lewis W, Zwilling B, Rinehart J. Characterization and modulation of human lymphokine (interleukin-2) activated killer cell induction. *Cancer Res* 1986; **46**: 2834–2838.
6. Lattime EC, Bykowsky MJ, Stutman O. Natural cytotoxic (NC) activity: a multi-lineage system regulated by interleukin-2. *Immunol Res* 1986; **5**: 5–15.
7. Zhang SR, Salup RR, Urias PE, Twilley TA, Talmadge JE, Herberman RB. Augmentation of NK activity and/or macrophage mediated cytotoxicity in the liver by biological response modifiers including human recombinant interleukin-2. *Cancer Immunol Immunother* 1986; **21**: 19–25.
8. Hellstrand K, Hermodsson S. Interleukin-2 can induce suppression of human natural killer cell cytotoxicity. *Clin Exp Immunol* 1989; **79**: 410–416.
9. Wahl SM, McCartney-Francis N, Hunt DA, Smith PD, Wahl LM, Katona IM. Monocyte interleukin-2 receptor gene expression and interleukin-2 augmentation of microbicidal activity. *J Immunol* 1987; **139**: 1342–1347.
10. Herrmann F, Cannistra SA, Levine H, Griffin JD. Expression of interleukin-2 receptors and binding of interleukin-2 by gamma interferon-induced human leukemic and normal monocytic cells. *J Exp Med* 1985; **162**: 1111–1116.
11. Hancock WW, Muller WA, Contran RS. Interleukin-2 receptors are expressed by alveolar macrophages during pulmonary sarcoidosis and are inducible by lymphokine treatment of normal human lung macrophages, blood monocytes, and monocyte cell lines. *J Immunol* 1987; **138**: 185–191.
12. Malkovsky M, Loveland B, North M, Asherson GL, Gao L, Ward P, Fiers W. Recombinant interleukin-2 directly augments the cytotoxicity of human monocytes. *Nature* 1987; **325**: 262–265.
13. Belosevic M, Nacy CA. IL-2, anti-IL-2 receptor antibody and activation of macrophages. *Cell Immunol* 1990; **128**: 635–640.
14. Gaulton GN, Eardley DD. Interleukin-2-dependent phosphorylation of interleukin-2 receptors and other T-cell membrane proteins. *J Immunol* 1986; **136**: 2470–2477.
15. Robb RJ, Rusk CM, Yodoi J, Greene WC. Interleukin-2 binding protein distinct from the Tac protein: analysis of its role in formation of high-affinity receptors. *Proc Natl Acad Sci USA* 1987; **84**: 2002–2006.
16. Bich-Thuy LT, Dukovich M, Peffer NJ, Nauci AS, Kehrl JH, Greene WC. Direct activation of human resting T-cells by IL2: the role of an IL2 receptor distinct from the Tac protein. *J Immunol* 1987; **139**: 1550–1556.
17. Campbell PA. The neutrophil, a professional killer of bacteria, may be controlled by T-cells. *Clin Exp Immunol* 1990; **79**: 141–143.
18. Dinarello CA. The pro-inflammatory cytokines interleukin-1 and TNF and treatment of the septic shock syndrome. *J Infect Diseases* 1991; **163**: 1177–1184.
19. Ferrante A, Thong YH. Optimal conditions for the simultaneous purification of mononuclear and polymorphonuclear phagocytes from human blood. *J Immunol Methods* 1980; **36**: 109–117.
20. Gmeylig-Meyling R, Waldermann TA. Separation of human blood monocytes and lymphocytes on a continuous percoll gradient. *J Immunol Methods* 1980; **33**: 1–9.
21. Thong YH, Currell JM. Development of a microassay technique for neutrophil adherence. *J Immunol Methods* 1983; **63**: 229–236.
22. Ferrante A, Beard J, Thong YH. Early decay of human neutrophil responsiveness following isolation from peripheral blood. *Clin Exp Immunol* 1980; **39**: 532–537.
23. Yamamura M, Boler J, Valdimarsson H. Phagocytosis measured as inhibition of uridine uptake by *Candida albicans*. *J Immunol Methods* 1977; **19**: 10–15.
24. Seow WK, Thong YH, McCormack JG, Ferrante A. Lymphokine-neutrophil interactions: opposite effects of interleukin-2 and tumour necrosis factor-beta (lymphotoxin) on human neutrophil adherence. *Int Arch Allergy Appl Immunol* 1988; **85**: 63–68.
25. Ferrante A, Rencis VO. Enhancement of base hexose-monophosphate shunt activity of human polymorphonuclear leukocytes by human γ-interferon. *Immunol Lett* 1989; **8**: 215–217.
26. Metcalf JA, Gallin JI, Nauseef WM, Root RK. *Laboratory Manual of Neutrophil Function.* New York: Raven Press, 1986; 147–148.
27. Hall ER, Papp AC, Seifert WE, Jr, Wu KK. Stimulation of endothelial cell prostacyclin formation by interleukin-2. *Lymphokine Res* 1986; **5**: 87–96.
28. Jablons D, Bolton E, Mertins S, Rubin M, Pizzo P, Rosenberg SA, Lotze M. Interleukin-2 based immunotherapy alters circulating neutrophil Fc receptor expression and chemotaxis. *J Immunol* 1990; **144**: 3630–3636.
29. Klempner MS, Noring R, Mier JW, Atkins MB. An acquired chemotactic defect in neutrophils from patients receiving interleukin-2 therapy. *N Engl J Med* 1990; **322**: 959–965.
30. Kowanko IC, Ferrante A. Interleukin-2 inhibits migration and stimulates respiratory burst and degranulation of human neutrophils *in vitro*. *Immunol Lett* 1987; **15**: 285–289.
31. Tauber AI, Karnad AB, Hartshorn KL, Myers JB, Smartz JH. Parameters of neutrophil activation: models of priming and deactivation. *Biochem Acute Allergic Reactions: Fifth Int Symp.* Alan R Liss, Inc., 1989; 297–309.
32. Lord R. Assessment of neutrophil function. *Med Lab Sci* 1989; **46**: 347–356.
33. Burdach S, Shatsky M, Wagenhorst B, Levitt L. Receptor specific modulation of myelopoiesis by recombinant DNA-derived interleukin-2. *J Immunol* 1987; **139**: 452–458.
34. Naldini A, Fleischmann WR, Zuhair JR, Ballas K, Klimpel KD, Klimpel GR. Interleukin-2 inhibits *in vitro* granulocyte–macrophage colony formation. *J Immunol* 1987; **139**: 1880–1884.

ACKNOWLEDGEMENTS. We wish to thank the Mater Public Hospital Immunology Laboratory for the monoclonal antibody staining and monocyte enumeration of mononuclear cell suspensions. Funding for this work was provided by the J. P. Kelly Mater Research Fund and the Mayne Bequest Fund, University of Queensland. We also thank Mary Herwig for the typing of this manuscript.

**Received 3 November 1992; accepted in revised form 7 December 1992**
